# Promoting Informed Decisions About Colorectal Cancer Screening in Older Adults (PRIMED Study): a Physician Cluster Randomized Trial

**DOI:** 10.1007/s11606-022-07738-4

**Published:** 2022-08-05

**Authors:** Karen Sepucha, Paul K. J. Han, Yuchiao Chang, Steven J. Atlas, Neil Korsen, Lauren Leavitt, Vivian Lee, Sanja Percac-Lima, Brittney Mancini, James Richter, Elizabeth Scharnetzki, Lydia C. Siegel, K. D. Valentine, Kathleen M. Fairfield, Leigh H. Simmons

**Affiliations:** 1 Division of General Internal Medicine, Massachusetts General Hospital Health Decision Sciences Center, Boston, MA USA; 2 Harvard Medical School, Boston, MA USA; 3 Center for Interdisciplinary Population and Health Research, Maine Medical Center, Portland, ME USA; 4 Division of Cancer Control and Population Sciences, National Cancer Institute, Bethesda, USA; 5 Division of Gastroenterology, Massachusetts General Hospital, Boston, MA USA; 6 Division of General Internal Medicine, Brigham and Women’s Hospital, Boston, MA USA

**Keywords:** shared decision-making, colorectal cancer screening, patient preferences/patient engagement, online training

## Abstract

**Background:**

For adults aged 76–85, guidelines recommend individualizing decision-making about whether to continue colorectal cancer (CRC) testing. These conversations can be challenging as they need to consider a patient’s CRC risk, life expectancy, and preferences.

**Objective:**

To promote shared decision-making (SDM) for CRC testing decisions for older adults.

**Design:**

Two-arm, multi-site cluster randomized trial, assigning physicians to Intervention and Comparator arms. Patients were surveyed shortly after the visit to assess outcomes. Analyses were intention-to-treat.

**Participants and Setting:**

Primary care physicians affiliated with 5 academic and community hospital networks and their patients aged 76–85 who were due for CRC testing and had a visit during the study period.

**Interventions:**

Intervention arm physicians completed a 2-h online course in SDM communication skills and received an electronic reminder of patients eligible for CRC testing shortly before the visit. Comparator arm received reminders only.

**Main Measures:**

The primary outcome was patient-reported SDM Process score (range 0–4 with higher scores indicating more SDM); secondary outcomes included patient-reported discussion of CRC screening, knowledge, intention, and satisfaction with the visit.

**Key Results:**

Sixty-seven physicians (Intervention *n*=34 and Comparator *n*=33) enrolled. Patient participants (*n*=466) were on average 79 years old, 50% with excellent or very good self-rated overall health, and 66% had one or more prior colonoscopies. Patients in the Intervention arm had higher SDM Process scores (adjusted mean difference 0.36 (95%CI (0.08, 0.64), *p*=0.01) than in the Comparator arm. More patients in the Intervention arm reported discussing CRC screening during the visit (72% vs. 60%, *p*=0.03) and had higher intention to follow through with their preferred approach (58.0% vs. 47.1, *p*=0.03). Knowledge scores and visit satisfaction did not differ significantly between arms.

**Conclusion:**

Physician training plus reminders were effective in increasing SDM and frequency of CRC testing discussions in an age group where SDM is essential.

**Trial Registration:**

The trial is registered on clinicaltrials.gov (NCT03959696).

**Supplementary Information:**

The online version contains supplementary material available at 10.1007/s11606-022-07738-4.

## INTRODUCTION

The United States Preventive Services Task Force (USPSTF) advises individualized decision-making for colorectal cancer (CRC) screening for people aged 76–85, due to the potentially limited benefit and higher risk of complications of CRC screening in this age group.^1^ For older patients undergoing surveillance colonoscopies, Tran et al.^2^ found a very low CRC incidence in patients 75 and older, but a higher risk for hospitalization with continued colonoscopy. The US Multi-Society Task Force guidelines also recommend the need to individualize surveillance decisions in adults 75 and older based on patients’ overall health, CRC risk, and preferences.^3^ Shared decision-making (SDM) is an approach to making medical decisions that incorporates the best available medical evidence, patients’ personal goals and preferences, and clinicians’ medical expertise to identify the best option.

Studies suggest that older patients are not routinely engaged in discussions around stopping testing, despite a high desire to be involved.^4^ Systematic reviews have identified several barriers to SDM implementation including time constraints, perceived lack of applicability, and lack of clinician support.^5,6^ A patient decision aid on CRC screening for older adults resulted in more appropriate screening intentions and behavior^7^; however, there are no clinician-directed interventions to promote SDM for this decision. Studies suggest a combination of evidence-based strategies to change physician behavior (such as persuasion, education, audit and feedback, and reminders) may be effective.^8,9^ There is a need for well-designed studies to examine clinician-directed strategies to promote SDM in routine care.^10^

The purpose of the PRomoting InforMEd Decisions about Cancer Screening in Older Adults (PRIMED) study was to examine the effectiveness of clinician training with a reminder (Intervention) to a reminder strategy alone (Comparator) for increasing shared decision-making for decisions about CRC testing. The training intervention was based on the recognition that medical education has not traditionally focused on how to engage patients in shared decision-making when evidence is limited. As a result, even with a reminder to discuss stopping testing with older patients, physicians may be hesitant to have these conversations. The reminder strategy assumed that physicians were experienced and capable of having a SDM discussion but needed a reminder to prompt the discussion. The trial tested hypotheses that patients seen by physicians in the Intervention arm would report higher levels of SDM, be more likely to discuss CRC testing, have greater knowledge, and have higher visit satisfaction than in the Comparator arm.

## METHODS

All study activities were approved by Mass General Brigham Institutional Review Board. The study was registered on Clinical Trials.gov (NCT03959696). Reporting followed the CONSORT guidelines.^11^ This work was funded by the Patient-Centered Outcomes Research Institute (PCORI) grant CDR-2017C3-9720.

### Design

The PRIMED study is a multi-site cluster randomized trial that enrolled primary care physicians and assigned them to Intervention or Comparator arms. Patients were surveyed after a visit with a participating physician to assess outcomes.

### Participants

#### Physician Sample

Primary care physicians from Internal Medicine and Family Medicine practices affiliated with five hospital networks—three academic medical centers and two community hospitals in the Northeast—were eligible if they had at least 20 patients aged 75–85 due for a CRC screening or surveillance in their panel. Residents and advance practice providers were excluded because, in most of these hospital networks, they do not carry their own patient panels.

#### Patient Sample

The study staff extracted a list of potentially eligible patients from each participating physician’s panel. Physicians reviewed their lists and excluded any patient based on the criteria in Table 1. Research staff reviewed physicians’ schedules to identify potentially eligible patients with an upcoming preventive care or scheduled follow-up appointment and confirmed eligibility before inviting them to participate.

**Table 1:** Eligibility Criteria for the Patient Sample

Eligible	Ineligible
• Adults, aged 76–85. • Due or overdue for colorectal cancer screening or surveillance test. Defined as no evidence of colonoscopy within recommended interval indicated on prior colonoscopy (e.g., 3, 5, 7, or 10 years); no evidence of barium enema, sigmoidoscopy, or CT colonography within 5 years; and no evidence of a stool-based test (FOBT, FIT or Cologuard) within the past year. • Attended non-urgent visit with participating physician during the enrollment period.	• Prior history of colon or rectal cancer • Prior colectomy • Physician exclusion due to major co-morbidity, limited cognitive capacity, or not being part of the patient panel. • Unable to read or write in English or Spanish. • Unable to consent for self.

Legend: *FOBT*, fecal occult blood test; *FIT*, fecal immunochemical test

### Interventions

#### SDM Skills Training

A 2-h online SDM communication skills training course included case studies and interactive exercises to simulate conversations with older patients about CRC testing (see Table 2 for details). The course was adapted from previous training sessions^12,13^ and incorporated features that have been shown to improve the effectiveness of continuing medical education (CME).^14^ Physician participants received 2 h of CME. They also received resources to use throughout the study, including a patient-facing education worksheet, the ability to submit cases and get feedback from study investigators, and an opportunity to complete an additional telephone-based simulated patient interaction to practice skills. Five email newsletters were sent to intervention arm physicians (about one per quarter during patient enrollment) summarizing key points from the challenging cases that were submitted by physician participants.

**Table 2:** Components of the Training Course and the Cases Used to Practice Skills

Course content	
Module 1	• Review clinical guidelines for colorectal cancer screening for patients aged 76–85 • Overview of shared decision-making and 7-step framework • Scoring two video vignettes (case: Mr. Sullivan) for elements of shared decision-making
Module 2	• In-depth description of 7 steps for shared decision-making with example scripts for each step: 1. Invite participation 2. Present options 3. Describe benefits and harms 4. Elicit goals and concerns 5. Facilitate deliberation 6. Support implementation 7. Involve trusted others • Scoring two video vignettes (case: Mrs. Turner) for elements of shared decision-making • Presentation of two risk calculators to estimate colorectal cancer risk and overall life expectancy
Module 3	Interactive case-based module where learners progress through four cases and determine (1) whether or not the patient is eligible/appropriate for the decision discussion and (2) for the two eligible cases (case: Mrs. Clark and Mr. Martinez) the learner progresses through the 7 steps of shared decision-making for each case, including accessing the online risk calculators to estimate CRC risk and life expectancy, with tips and feedback.
Case descriptions	
Mr. Sullivan	81-year-old man with heart disease and arthritis with prior spine and hip surgery. He had an abnormal polyp 6 years ago and is overdue for follow-up colonoscopy. He asks whether he really needs one at his age. His wife wants him to get one.
Mrs. Turner	76-year-old woman, recently moved to town to be closer to her daughter. She is new to the practice, is very healthy, but has never been screened before (prior PCP notes mention she declined colonoscopy).
Ms. Clark	83-year-old woman with rheumatoid arthritis who is extremely frail and lives alone. Her mother was diagnosed with colon cancer at 90. All of her past colonoscopies were normal. Her last one was at age 73 and she wants to schedule another one.
Mr. Martinez	78-year-old man who has hypertension and high cholesterol. He is moderately active and has no family history of CRC. His first colonoscopy (age 56) was normal, his second (age 66) removed single tubular adenoma, and his third (age 71) was normal.

#### Reminders

Research staff sent an email or electronic health record (EHR) message (per physician preference) to the participating physician 2–3 days prior to a visit with an eligible patient. The message encouraged the physician to have a conversation with the patient about whether to continue CRC testing and included the patient’s last CRC test and date, if applicable.

### Study Procedures

#### Randomization and Blinding

Eligible physician participants were grouped into strata based on self-reported gender, years in practice, prior exposure to SDM training (self-reported by physicians at enrollment), and site. Within each stratum, we assigned physicians to one of two arms (Intervention or Comparator) using a random number generator. It was not possible to blind physician participants to study interventions; however, no details were given regarding the content of the arms (only an estimate of the time required for each arm). Physicians were aware that their patients would be surveyed but were not given details on the content of the survey. Patients were blinded to the study arms. The PIs and the project manager were not blinded to physicians’ assignment as they needed to arrange access to training courses. Research staff who entered the patient survey data into REDCap and the biostatistician conducting the analyses were blinded.

#### Study Protocol

We enrolled physicians from May 2019 through August 2019. Physicians were notified of the study at practice meetings, presentations, newsletters, and individual invitations. Interested physicians were screened to confirm eligibility and indicated consent by sending an email confirming their intention to join.

From October 28, 2019, through March 13, 2020, eligible patients were mailed a study invitation and an information sheet describing the study 2–3 weeks before a visit. The cover letter included information for patient participants to opt out by calling or sending a postcard to the study team. Shortly after the scheduled visit, staff confirmed that the visit occurred and sent a packet including a $5 incentive to all patients who had not opted out. The staff made up to three reminder calls and sent a reminder packet to patient non-responders about 4 weeks after the initial packet. Due to the low-risk nature of the study, patient consent was implied by returning the survey.

The study was suspended on March 13, 2020, due to restrictions imposed by the COVID-19 pandemic. Enrollment resumed at 4 of the 5 networks on May 26, 2020, and at the 5^th^ network on September 8, 2020. When enrollment resumed, the survey protocol was changed to accommodate COVID-era changes to visits. Staff mailed the study packet after the patient’s visit rather than before due to the large amount of rescheduling of in-person visits to virtual visits that occurred shortly before appointments. The same reminder protocol was followed. Enrollment closed on April 2, 2021.

### Patient Survey Outcomes and Measures

- Shared Decision-Making Process Scale (primary outcome): this 4-item measure assessed the discussion of (1) stopping screening as an option, (2) reasons to screen, (3) reasons not to screen, and (4) patients’ screening preferences. Individual items were summed to generate a total score (0–4), with higher scores indicating greater shared decision-making. Patients who indicated no discussion received a score of 0. This brief measure has strong evidence of acceptability, feasibility, reliability, and validity.^15–17^ Although there is no clearly established clinical meaningful difference, effect sizes from 0.39SD to 0.88SD are found when comparing sites with and without formal decision support.^17^
- Knowledge: seven multiple-choice knowledge items, adapted from the Colorectal Cancer Screening Decision Quality Instrument, were scored and summed to calculate a total knowledge score (0–100%).^18^
- Patient’s screening preference: a single item assessed patients’ preferred approach to screening with responses: colonoscopy, stool-based test, no screening, and not sure.
- Patient’s screening intention: one item assessed how likely the patient was to follow through with their preferred approach on a 5-point scale from Definitely will to Definitely will not.
- CRC discussion: one item assessed whether CRC screening was discussed during the visit (yes/no) and if discussed, how much time was spent (<2 min, 2–5 min, > 5 min).
- Satisfaction: one item asked “Overall, how satisfied were you with the visit” on a 4-point scale from Extremely satisfied to Not at all satisfied.

Patients also self-reported overall physical and mental health *(*PROMIS Scale v1.2-Global Health Physical 2a (poor to excellent)^19^, family history of colorectal cancer, personal history of prior polyp removal, health literacy (Single-Item Literacy Screener)^20,21^, race, ethnicity, marital status, and education.

Physicians completed a short baseline survey to collect demographics and prior SDM training experience.

### Sample Size

The study was powered to detect a small to medium effect size difference in the primary outcome, SDM process score.^17^ With 500 surveys, assuming an intraclass correlation coefficient of 0.03, the effective patient sample size was estimated at 394, which would enable detection of a difference of 0.28 standard deviations with 80% power and a two-sided significance level of 0.05.

### Analysis

Responders and non-responders were compared to examine potential non-response bias. Patient sample characteristics were compared between arms since randomization occurred at the physician level. Multivariable regression models were used to adjust for potential effects of any unbalanced variables. The hypotheses were evaluated using an intention-to-treat approach and patient outcomes were analyzed based on their physicians’ assigned arm regardless of whether the physician completed the training, received the reminder, or discussed CRC screening.

We tested hypotheses that patients seen by physicians in the Intervention arm would report higher SDM Process scores, be more likely to discuss CRC screening, have greater knowledge, and have higher visit satisfaction than patients of physicians in the Comparator arm. We used a linear regression model with Generalized Estimating Equations (GEE) techniques to account for the patients-within-physicians data structure and compare the continuous outcomes (SDM Process and Knowledge scores) between arms.^22^ We used logistic regression models with GEE for binary outcomes, ordinal regression for ordered categorial outcomes, and multinomial regression for categorical outcomes (e.g., screening preference). Models included study arm, patient age, patient sex, prior screening (yes/no), physician age, physician gender, physician years since training, and hospital network.

#### Heterogeneity of Treatment Effects (HTE)

The pre-specified HTE analysis explored the interaction between the study arm and different factors on outcomes. Physician factors included (1) hospital network, (2) gender, (3) age, (4) years in practice, and (5) prior experience with SDM training. Patient factors included (1) sex, (2) age, (3) prior screening history, and (4) overall health. Linear or logistic regression models with the GEE approach were used to test interactions between study arms and these factors. Due to the exploratory nature of the HTE analysis, we reported treatment effects in each subpopulation when the significance level for the interactions between intervention and these factors was ≤0.1.

### Patient and Public Involvement in the Research Study

Four patient advisors actively participated on the study team. They attended meetings and provided feedback on study design, training content, communication and messaging to patient participants, and selection of outcomes.

## RESULTS

**Figure 1:**
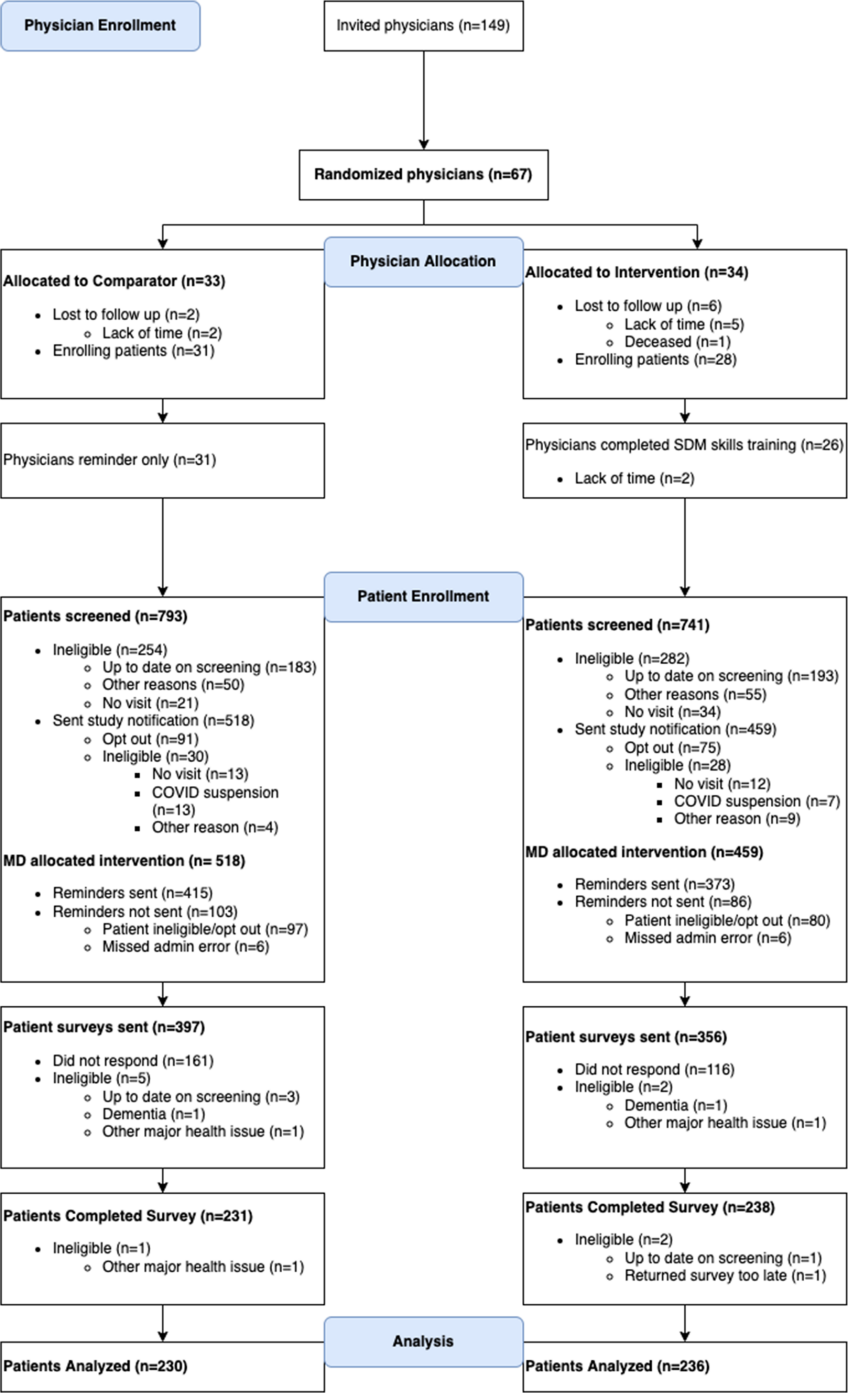
CONSORT diagram for physician and patient enrollment.

Sixty-seven physicians enrolled (67/149, 45% consent rate) from 35 clinics within the 5 networks and were randomly assigned to an arm based on strata. The physicians were similar in age, gender, network, years in practice, and enrolled patient volume across arms (see Table 3). Figure 1 shows the CONSORT diagram for the physicians and for the patients in the study.

**Table 3:** Physician Characteristics for the Study Arms

	Analytic sample	
Variable	Intervention *N*=28	Comparator *N*=31
Age: mean (SD)	53.1 (10.0)	52.4 (9.0)
Female: *n* (%)	14 (50.0%)	16 (51.6%)
Years in practice: mean (SD)	22.4 (10.9)	20.9 (9.6)
Number of enrolled patients: median (range)	8 (0, 25)	6 (1, 20)
Prior SDM training: *n* (%)	8 (28.6%)	8 (25.8%)
Academic practice (vs. community practice): *n* (%)	18 (64.3%)	19 (61.3%)

*SD *standard deviation, *SDM* shared decision making

We received 469/753 (62.3%) patient surveys and found three to be ineligible, resulting in an analytic sample of 466 (Fig. 1). Patient responders were slightly younger, less likely to be female, and more likely to have had a prior screening test than non-responders (see supplemental eTable 1). The patient characteristics were generally similar between arms (Table 4); however, the Intervention arm had a higher percentage of women (58.5% vs. 48.3%, *p*=0.03) and a higher percentage of patients with a prior screening test (87.7% vs. 80.4%, *p*=0.04) than the Comparator arm.

**Table 4:** Patient Characteristics for the Study Arms

	Analytic sample	
Variable	Intervention *N*=236	Comparator *N*=230
Age: mean (SD)	79.5 (2.8)	79.2 (2.8)
Female: *n* (%)	138 (58.5)	111 (48.3)
Prior test: *n* (%)		
Colonoscopy	159 (67.4)	152 (66.1)
Stool-based test	48 (20.3)	33 (14.3)
None on record	29 (12.3)	45 (19.6)
Family history of colorectal cancer: *n* (%)	47 (20.3)	42 (19.0)
Prior polyps removed: *n* (%)	115 (50.7)	107 (47.6)
Academic practice (vs. community practice), *n* (%)	135 (57.2)	125 (54.3)
Marital status		
Married/living with partner	135 (57.2)	136 (59.1)
Widowed	54 (22.9)	47 (20.4)
Separated/divorced	32 (13.6)	25 (10.9)
Single (never married)	11 (4.7)	14 (6.1)
Other/missing	4 (1.7)	8 (3.5)
High health literacy	198 (85.3)	189 (82.5)
Physical health (excellent or very good)	123 (53.7)	115 (50.7)
Race/ethnicity White, non-Hispanic: *n* (%)	218 (92.4)	215 (93.5)
Education: *n* (%)		
High school graduate or less	63 (26.7)	65 (28.3)
Some college or 2-year degree	39 (16.5)	53 (23.0)
4-year college graduate	42 (17.8)	40 (17.4)
More than 4-year degree	89 (37.7)	68 (29.6)

*SD* standard deviation

For the primary outcome, the Intervention arm had higher SDM Process scores than the Comparator arm (adjusted mean difference 0.36 (95%CI (0.08, 0.64), *p*=0.01). Intervention arm patients were significantly more likely to report that the doctor talked “a lot” or “some” about reasons to screen and asked about their preferences (Table 5). Furthermore, patients were more likely to report that the physician discussed stool-based tests (51.1% vs. 27.3%, *p*<0.001).

**Table 5:** Shared Decision-Making Process Items and Total Score

	Intervention* N= 232	Comparator* N= 222	*P*
Talked about no further testing as an option	91 (39.2%)	72 (32.4%)	0.19
Talked about reasons to screen			0.02
A lot	16 (6.9%)	16 (7.2%)	
Some	93 (40.1%)	51 (23.0%)	
A little	41 (17.7%)	46 (20.7%)	
Not at all	82 (35.3%)	109 (49.1%)	
Talked about reasons not to screen			0.66
A lot	11 (4.75%)	11 (5.0%)	
Some	54 (23.3%)	39 (17.6%)	
A little	44 (19.0%)	30 (13.5%)	
Not at all	123 (53.0%)	142 (64.0%)	
Asked patient preferences	145 (62.5%)	95 (42.8%)	<0.001
Total score, mean (SD)	1.5 (1.2)	1.1 (1.2)	0.001

*Limited to those with complete data on all four items

Intervention arm patients also reported more CRC screening discussions (72% vs. 60%, adjusted OR 1.76 [1.07, 2.91] *p*=0.03). When CRC screening was discussed, the amount of time spent did not vary between arms (*p*=0.75). About half of patients reported 2–5 min (54.7% Intervention and 56.5% Comparator), less than 2 min (21.2% Intervention and 26.1% Comparator), or more than 5 min (24.1% Intervention and 17.4% Comparator).

Overall, knowledge scores did not differ between arms (63.0% vs. 61.0%, *p*=0.36). Nearly all patient participants understood the main benefit of screening (91.9% and 93.5%) and that most colon cancers start as a polyp (94.1% and 87.4%). Fewer understood that testing and no testing are both reasonable options for people 76–85 years old (61.4% and 51.7%), that stool testing is usually done every year (55.5% and 57.4%), and that serious complications with colonoscopy are rare (66.5% and 64.8%). Full knowledge results are in supplemental eTable 2.

Patients’ preferred approach did not differ between arms (*p*=0.36). Among respondents, 34.5% preferred stool-based tests, 25.2% colonoscopy, 20.6% no further screening and, 17.0% not sure. Patients in the Intervention arm were more likely to report that they “definitely” intended to follow through with their preferred approach to screening than those in the Comparator arm (58.0% vs. 47.1%, *p*=0.02).

More patients reported being “extremely satisfied” with the visit in the Intervention arm than in the Comparator arm, although this difference was not statistically significant (67.5% vs. 56.0%, *p*=0.10).

In the planned heterogeneity analyses, patients in the Intervention arm had a larger increase in SDM scores over the Comparator for patients 80–85 versus those 76–79 (adjusted mean difference (aMD) 0.49 vs. 0.19 points), for male versus female patients (aMD 0.64 vs. 0.15 points), for those without a family history (aMD 0.47 vs. 0.05 points), for those without prior polyps (aMD 0.51 vs. 0.21 points), and for physicians with < 25 years in practice versus those with ≥ 25 years (aMD 0.69 vs. 0.18 points). Supplemental eTable 3 has the full HTE results.

## DISCUSSION

This study addresses an important gap in our understanding of how to promote shared decision-making about continuing or completing CRC screening in older adults. The Intervention, a brief training plus reminders, resulted in higher patient-reported SDM scores and more frequent discussions about CRC screening compared to the reminder alone. The Intervention arm physicians were more likely to ask patients about their preferences and to discuss stool-based tests than those in the Comparator. The intervention appeared particularly effective at improving SDM scores for older patients, male patients, patients without prior polyps or family history of CRC, and patients seen by physicians with fewer years of experience. There was no significant difference in patients’ level of knowledge or visit satisfaction between arms, but patients seen by physicians in the Intervention arm did report higher intention to follow through with their preferred approach.

In this study, a majority of patients in both arms reported that CRC screening was discussed during the visit. Prior studies have found that CRC screening decisions among older adults are not always deliberate or explicit, with about 50% of patients reporting the topic was never brought up.^4^ Although there was no usual care arm, comparing the rates of discussion in this study to the literature strongly suggests a positive effect of the reminder in prompting discussions. Systematic reviews typically find little impact of CME on physician’s behavior, and studies suggest that multiple strategies, including reminders, are needed to promote physician behavior change.^23,24^ Patients of physicians in the SDM training group reported significantly more screening discussions and greater SDM scores, suggesting these strategies enhanced the impact of the reminder component of the intervention. Our study thus supports both current expert consensus and accumulating evidence on the value of SDM skills training for physicians.^25,26^

Our findings also highlight the challenges involved in integrating SDM in routine primary care. The absolute SDM scores for both arms were low. This result tracks with other studies examining SDM in CRC screening that find little to no SDM in these conversations.^15,16^ The level of CRC risk, whether due to family history or prior polyps, may influence the discussion of options. Subgroup analyses found that compared to Comparator physicians, Intervention physicians were more likely to engage in SDM with patient groups without risk factors (i.e., no family history, no prior polyps). The training had less impact on physicians with more than 25 years of experience, suggesting that changing practice to promote SDM may require different interventions for this group.

The low SDM scores may also be due to the very brief conversations, as the majority reported less than 5 min spent discussing CRC screening. It is not likely that physicians will find much more time; and as a result, it may be necessary to involve other care team members or use tools such as patient decision aids to achieve better outcomes. Patient decision aids are effective in increasing knowledge for CRC screening decisions generally^27^ and there is one existing study using a decision aid for decisions about discontinuing CRC screening for older patients.^7^ Studies examining the effectiveness of interventions designed to implement SDM find that targeting both physicians and patients appear to be most effective.^10^ The training and reminders tested here were helpful but not sufficient; there is still substantial room for improvement in implementing SDM.

These findings should be considered in the context of the limitations of this study. First, a significant portion of study visits (60%) happened during the COVID-19 pandemic, which caused significant disruptions to visits and care. The Intervention arm SDM training was completed in the summer of 2019, and any COVID impact on clinical operations affected both arms equally. We did not find a significant interaction between outcomes in pre- and post-COVID periods (data not shown). Second, the study did not include a usual care arm because we felt that it would have had too few discussions, and as a result, we are not able to provide an estimate of the impact of the Intervention or Comparator over usual care. Third, the enrolled patient sample had limited racial and ethnic diversity, which limits the generalizability of these results. Fourth, randomization at the physician level resulted in more female patients and more patients reporting prior screening in the Intervention arm, and all analyses adjusted for those variables. Finally, all physicians knew that their patients were being surveyed, which may have led them to discuss CRC testing more often than usual.

Our findings have implications for clinical practice. Most EHRs have automatic flags or alerts to prompt initiation of cancer screening or address overdue screening. However, the automatic alerts for CRC screening are removed when patients reach age 76. Automating the reminder for a SDM conversation about CRC testing for older adults may be a feasible and scalable notification strategy. Furthermore, this notification would benefit from focused clinician training and tools to help clarify benefits and risks to support appropriate recommendations and more efficient discussions. Future studies are also warranted to examine the role of patient decision aids and the involvement of other clinic staff in CRC testing discussions to enhance feasibility and effectiveness. Just as patients deserve thoughtful conversations about when and how to start cancer screening in mid-life, it is time for explicit, nuanced conversations with patients about the completion of cancer screening in later life.

## Supplementary Information

ESM 1 (DOCX 29 kb)

## Data Availability

After the study results have been published, de-identified data sets will be deposited in an open access service, ICPSR (https://www.icpsr.umich.edu/icpsrweb/). Before then, the datasets will be available from the corresponding author on reasonable request.
