# Supplementary material for: Promoting Informed Decisions About Colorectal Cancer Screening in Older Adults (PRIMED Study): a Physician Cluster Randomized Trial
Source: J Gen Intern Med. 2022 Aug 5;38(2):406–13. doi: 10.1007/s11606-022-07738-4 (PMC9362387; doi:10.1007/s11606-022-07738-4)
Supplement: Supplementary file 1 — (DOCX 29 kb) [file 11606_2022_7738_MOESM1_ESM.docx]

Appendix eTable 1: Responders versus non responders (including those who opted out prior to the visit)

|  | | All | | Intervention | | Comparator | |
| --- | --- | --- | --- | --- | --- | --- | --- |
|  |  | Responder | | Responder | | Responder | |
|  |  | No | Yes | No | Yes | No | Yes |
| N | | 440 | 466 | 188 | 236 | 252 | 230 |
| Age *^,†^ | Mean (SD) | 79.8 (2.8) | 79.4 (2.8) | 79.8 (2.8) | 79.6 (2.8) | 79.8 (2.8) | 79.2 (2.8) |
| Gender ^†^ |  | 259 | 249 | 114 | 138 | 145 | 111 |
| Female | N |  |  |  |  |  |  |
|  | % | 58.9 | 53.4 | 60.6 | 58.5 | 57.5 | 48.3 |
| Male | N | 181 | 217 | 74 | 98 | 107 | 119 |
|  | % | 41.1 | 46.6 | 39.4 | 41.5 | 42.5 | 51.7 |
| Language |  | 437 | 463 | 187 | 234 | 250 | 229 |
| English | N |  |  |  |  |  |  |
|  | % | 99.3 | 99.4 | 99.5 | 99.2 | 99.2 | 99.6 |
| Spanish | N | 3 | 3 | 1 | 2 | 2 | 1 |
|  | % | 0.7 | 0.6 | 0.5 | 0.8 | 0.8 | 0.4 |
| Site |  | 169 | 179 | 66 | 89 | 103 | 90 |
| 1 | N |  |  |  |  |  |  |
|  | % | 38.4 | 38.4 | 35.1 | 37.7 | 40.9 | 39.1 |
| 2 | N | 39 | 25 | 25 | 17 | 14 | 8 |
|  | % | 8.9 | 5.4 | 13.3 | 7.2 | 5.6 | 3.5 |
| 3 | N | 89 | 108 | 48 | 59 | 41 | 49 |
|  | % | 20.2 | 23.2 | 25.5 | 25.0 | 16.3 | 21.3 |
| 4 | N | 85 | 98 | 26 | 42 | 59 | 56 |
|  | % | 19.3 | 21.0 | 13.8 | 17.8 | 23.4 | 24.3 |
| 5 | N | 58 | 56 | 23 | 29 | 35 | 27 |
|  | % | 13.2 | 12.0 | 12.2 | 12.3 | 13.9 | 11.7 |
| Prior Test ^*, †^ |  | 249 | 311 | 116 | 159 | 133 | 152 |
| Procedures | N |  |  |  |  |  |  |
|  | % | 56.6 | 66.7 | 61.7 | 67.4 | 52.8 | 66.1 |
| Stool-based tests | N | 82 | 81 | 41 | 48 | 41 | 33 |
|  | % | 18.6 | 17.4 | 21.8 | 20.3 | 16.3 | 14.3 |
| None | N | 109 | 74 | 31 | 29 | 78 | 45 |
|  | % | 24.8 | 15.9 | 16.5 | 12.3 | 31.0 | 19.6 |

*: p<0.05 for overall sample; †: p<0.05 for comparator.

Appendix eTable 2: Results of the knowledge items

| Item | Intervention  N=236 | Comparator  N=230 |
| --- | --- | --- |
| What is the main benefit of testing for colon cancer?   - Help prevent irritable bowel syndrome - Help one live longer - Help prevent cancer and find cancer early **(correct)** - Help one lose weight | 217 (91.9%) | 215 (93.5%) |
| For people aged 76-85, what do national experts recommend for testing for colon cancer?   - Routine tests for all - No further testing for all - Testing and **not** testing are both recommended options **(correct)** | 145 (61.4%) | 119 (51.7%) |
| How do most colon cancers start?   - As a tear in the colon - As a polyp in the colon **(correct)** - As a result of constipation - As a hemorrhoid | 222 (94.1%) | 201 (87.4%) |
| For people at average risk for colon cancer, which test is usually done every year?   - Colonoscopy - Stool-based test **(correct)** - Sigmoidoscopy | 131 (55.5%) | 132 (57.4%) |
| How often do major problems, such as serious bleeding or a tear in the colon, happen as a result of a colonoscopy?  □ Usually  □ Sometimes  □ Rarely **(correct)**  □ Never | 157 (66.5%) | 149 (64.8%) |
| Out of every 100 people aged 76-85, about how many will get colon cancer some time during the rest of their lives?   - 2  **(correct)** - 12 - 22 - 32 | 100 (42.4%) | 93 (40.4%) |
| For people who have had a normal colonoscopy in the past, which test is most effective for future screening?   - Stool-based tests - Colonoscopy - They are both about the same **(correct)** | 42 (17.8%) | 47 (20.4%) |

Appendix eTable 3: Results of Heterogeneity analyses for SDM Process scores

|  | n | Intervention | n | Comparator | Difference  (95% CI) | p | Interaction p |
| --- | --- | --- | --- | --- | --- | --- | --- |
| Patient Age  < 80 | 119 | 1.4 (1.2) | 122 | 1.1 (1.2) | 0.19 (-0.11, 0.50) | 0.22 | 0.08 |
| ≥ 80 | 113 | 1.5 (1.2) | 100 | 1.0 (1.2) | 0.49 (0.17, 0.82) | 0.003 |  |
| Patient Gender  Female | 134 | 1.4 (1,.2) | 106 | 1.1 (1.3) | 0.15 (-0.16, 0.47) | 0.34 | 0.09 |
| Male | 98 | 1.6 (1.2) | 116 | 1.0 (1.2) | 0.64 (0.27, 1.00) | <0.001 |  |
| Global Health Score  Excellent/Very good | 120 | 1.5 (1.2) | 110 | 1.2 (1.3) | 0.24 (-0.10, 0.59) | 0.16 | 0.22 |
| Good/fair/poor | 105 | 1.4 (1.2) | 109 | 0.9 (1.1) | 0.56 (0.21, 0.90) | 0.002 |  |
| Prior screening  Colonoscopy | 157 | 1.4 (1.2) | 149 | 1.2 (1.3) | 0.24 (-0.07, 0.55) | 0.13 | 0.17 |
| Stool based test | 46 | 1.5 (1.2) | 32 | 0.6 (0.9) | 0.87 (0.50, 1.24) | <0.001 |  |
| None | 29 | 1.4 (1.2) | 41 | 1.1 (1.2) | 0.49 (0.02, 0.96) | 0.04 |  |
| Family history |  |  |  |  |  |  |  |
| Yes | 46 | 1.1 (1.1) | 40 | 1.4 (1.3) | 0.05 (-0.31, 0.41) | 0.78 | 0.01 |
| No | 181 | 1.6 (1.2) | 173 | 1.0 (1.2) | 0.47 (0.19, 0.76) | 0.001 |  |
| Prior polyp removed |  |  |  |  |  |  |  |
| Yes | 113 | 1.4 (1.2) | 106 | 1.1 (1.2) | 0.21 (-0.14, 0.57) | 0.24 | 0.085 |
| No | 110 | 1.6 (1.2) | 111 | 1.0 (1.2) | 0.51 (0.19, 0.84) | 0.002 |  |
| Physician gender  Female | 93 | 1.3 (1.2) | 98 | 0.9 (1.2) | 0.40 (0.01, 0.73) | 0.04 | 0.66 |
| Male | 139 | 1.5 (1.2) | 124 | 1.2 (1.2) | 0.36 (-0.06, 0.78) | 0.09 |  |
| Physician age  <55 | 110 | 1.8 (1.2) | 153 | 1.1 (1.2) | 0.60 (0.32, 0.87) | <0.001 | 0.65 |
| ≥55 | 122 | 1.5 (1.2) | 69 | 1.1 (1.3) | 0.10 (-0.29, 0.49) | 0.62 |  |
| Site  1 | 88 | 1.2 (1.2) | 86 | 0.9 (1.2) | 0.28 (-0.16, 0.72) | 0.22 | 0.75 |
| 2 | 17 | 1.9 (1.5) | 8 | 1.0 (1.4) | 1.49 (0.94, 2.04) | <0.001 |  |
| 3 | 56 | 1.7 (1.2) | 46 | (1.5 (1.1) | 0.50 (0.31, 0.69) | <0.001 |  |
| 4 | 42 | 1.5 (1.2) | 56 | 0.8 (1.2) | 0.87 (0.09, 1.65) | 0.03 |  |
| 5 | 29 | 1.5 (1.2) | 26 | 1.5 (1.4) | 0.20 (-0.71, 1.11) | 0.66 |  |
| Years in practice  <25 | 117 | 1.7 (1.2) | 116 | 1.0 (1.2) | 0.69 (0.34, 1.04) | <0.001 | 0.03 |
| ≥25 | 115 | 1.2 (1.2) | 106 | 1.2 (1.2) | 0.18 (-0.21, 0.56) | 0.35 |  |
| COVID impact  Before shut down | 92 | 1.7 (1.2) | 80 | 1.3 (1.2) | 0.50 (0.07, 0.92) | 0.02 | 0.90 |
| After re-opening | 140 | 1.3 (1.2) | 142 | 1.0 (1.2) | 0.24 (-0.05, 0.54) | 0.11 |  |
